# Improved Understanding of the High Shear Wet Granulation Process under the Paradigm of Quality by Design Using *Salvia miltiorrhiza* Granules

**DOI:** 10.3390/pharmaceutics11100519

**Published:** 2019-10-09

**Authors:** Yi Zhang, Brian Chi-Yan Cheng, Wenjuan Zhou, Bing Xu, Xiaoyan Gao, Yanjiang Qiao, Gan Luo

**Affiliations:** 1 School of Chinese Material Medica, Beijing University of Chinese Medicine, Beijing 100029, China; yizhang714@163.com (Y.Z.); zhouwenjuan5113@163.com (W.Z.); btcm@163.com (B.X.); gaoxiaoyan0913@sina.com (X.G.); yjqiao@263.net (Y.Q.); 2 College of Professional and Continuing Education, Hong Kong Polytechnic University, Hong Kong 999077, China; brichian@hotmail.com; 3 Interdisciplinary Research Center on Multi-Omics of Traditional Chinese Medicine, Beijing University of Chinese Medicine, Beijing 102400, China; 4 Beijing Key Laboratory for Production Process Control and Quality Evaluation of Traditional Chinese Medicine, Beijing Municipal Science & Technology Commission, Beijing 102400, China

**Keywords:** high shear wet granulation, quality by design, *Salvia miltiorrhiza* granules, scale-up, regime map

## Abstract

Background: High shear wet granulation (HSWG) is a shaping process for granulation that has been enhanced for application in the pharmaceutical industry. However, study of HSWG is complex and challenging due to the relatively poor understanding of HSWG, especially for sticky powder-like herbal extracts. Aim: In this study, we used *Salvia miltiorrhiza* granules to investigate the HSWG process across different scales using quality by design (QbD) approaches. Methods: A Plackett–Burman experimental design was used to screen nine granulation factors in the HSWG process. Moreover, a quadratic polynomial regression model was established based on a Box–Behnken experimental design to optimize the granulation factors. In addition, the scale-up of HSWG was implemented based on a nucleation regime map approach. Results: According to the Plackett–Burman experimental design, it was found that three granulation factors, including salvia ratio, binder amount, and chopper speed, significantly affected the granule size (*D*_50_) of *S. miltiorrhiza* in HSWG. Furthermore, the results of the Box–Behnken experimental design and validation experiment showed that the model successfully captured the quadratic polynomial relationship between granule size and the two granulation factors of salvia ratio and binder amount. At the same experiment points, granules at all scales had similar size distribution, surface morphology, and flow properties. Conclusions: These results demonstrated that rational design, screening, optimization, and scale-up of HSWG are feasible using QbD approaches. This study provides a better understanding of HSWG process under the paradigm of QbD using *S. miltiorrhiza* granules.

## 1. Introduction

Granulation, the process of particle enlargement by agglomeration techniques, is routinely used across several industries to transform the properties of compressible powder blends [1]. Among all the powder agglomeration processes, high shear wet granulation (HSWG), which is a typical batch process, is one of the most commonly used techniques. HSWG is a good choice for many pharmaceutical applications to improve flow properties, compressibility, uniformity, and dosing accuracy for subsequent processing [2]. The advantages of HSWG include a short production time and simultaneous mixing and granulation [3]. Improving certain powder properties, HSWG monitoring, control, and scale-up are complicated because the granulation mechanism is unclear and too many factors affect the properties of the granules [4,5]. Moreover, there is a persistent problem known as “overgranulation” in HSWG, which results in substantial loss of the ability of granulated powders to be compressed into intact tablets [6]. Therefore, comprehensive understanding of the science and engineering of HSWG for controlling factors affecting the quality of the granules, such as granule size distribution, remains a serious challenge.

Quality by design (QbD) is a systematic approach to pharmaceutical development that begins with predefined objectives and emphasizes the understanding of product and process in addition to process control, based on sound science and quality risk management [7]. It has been applied to analytical methods, such as liquid chromatography [8], micellar electrokinetic chromatography [9], and mass spectrometry [10,11]. Regarding pharmaceutical manufacturing processes, to offer an understanding of how formulation and process variables influence the final drug product quality, QbD involves the application of fit-for-purpose engineering models to the formulation design, process optimization, and scale-up of active pharmaceutical ingredient (API) production and preparation manufacturing processes [12,13,14]. Notably, some researchers applied QbD principles to HSWG process development to achieve high-quality end products [15,16]. However, these reports focused on one or two aspects (including formulation design, process investigation, and scale-up of the HSWG process) in lieu of a comprehensive evaluation of these three perspectives together for the same pharmaceutical API in a HSWG process using QbD approaches [17,18]. Therefore, the combination of formulation design, process investigation, and rational scale-up to systematically study the HSWG process within the framework of QbD is urgently needed. 

*Salvia miltiorrhiza* (“danshen” in Chinese) is a Chinese medicine commonly used to promote blood circulation, remove blood stasis, alleviate pain, and relieve anxiety [19,20]. It has been widely applied clinically in the treatment of obstructive cerebrovascular diseases [21], atherosclerosis [22], coronary heart disease [23], and other cardiovascular diseases [24,25]. Furthermore, many different preparations of *S. miltiorrhiza* can be found, including compounded danshen dripping pills [26], danhong injection [27], danshen injection [28], danshen tablets [29], and danshen granules [30]. Moreover, *S. miltiorrhiza* extract is a high-value raw pharmaceutical material commonly used in Chinese patented medicine production. Danshen granules are also a common household medication for cardiovascular and cerebrovascular patients. However, in the manufacturing process of granules, there remains many unsolved problems, including caking [31], overgranulation [32], and non-uniform distribution of binder [33], all of which could affect the granule quality and restrict the further evolution of *S. miltiorrhiza* granules. Additionally, as a raw material, *S. miltiorrhiza* extracts have high viscosity, representing a big problem for the granulation process. Thus, there is great potential for improving the quality of *S. miltiorrhiza* granules from the aspects of formulation design, process investigation, and rational scale-up.

The purpose of this study was to obtain improved knowledge and understanding of the HSWG process using *S. miltiorrhiza* as the API model. Moreover, we also proposed to investigate how the formulation variables and process parameters affect the granule attributes, and how the process parameters could be tuned to gain similar granules across different scales. We also examined the application of design of experiment (DoE) to screen, optimize, validate, and carry out dimensional analysis-based scale-up of the HSWG process within the framework of QbD.

## 2. Experiment

### 2.1. Materials

*S. miltiorrhiza* extract powder (Batch No. 131210) was purchased from Xi’an Hong Sheng Biotechnology Co., Ltd (Xi’an, China). Dextrin (Batch No. 20140220) was obtained from Sinopharm Chemical Reagent Co., Ltd. (Beijing, China). The granule sizes (*D*_50_) of *S. miltiorrhiza* extract powder and dextrin were 20.45 and 12.08 μm, respectively. The angles of repose of these two powders were 48.24° and 45.25°, respectively. These two powders were used as the solid material. Ethanol of analytical grade from Beijing Chemical Works (Beijing, China) was diluted as the binder solution.

### 2.2. Instrumentation and Characterization

#### 2.2.1. Granulation Procedures

A specially constructed lab-scale HSWG machine (SHK-4, Xi’an Run Tian Pharmaceutical Machinery Co., Ltd., Xi’an, China) was used in this study. The geometrical parameters of the three replaceable stainless steel working vessels were as follows: the inner diameter, impeller radius, and chopper group for the 1-L scale vessel were 140 mm, 70 mm, and 2, respectively. For the 2-L scale vessel, the above parameters were 190 mm, 95 mm, and 3, respectively. Similarly, the parameters for the 4-L scale vessel were 240 mm, 120 mm, and 4, respectively. The depth of all three vessels was 165 mm. A replaceable three-bladed impeller made of mild steel was centrally located on the base of the vessel. Each blade of the impeller was identical, with the leading edge inclined by about 15°. A chopper was installed on the sidewall. The rotational speeds of the impeller and chopper were electronically controlled and could be adjusted from 0 to 1200 rpm and from 0 to 2900 rpm, respectively. The total weights of the solid powders (*S. miltiorrhiza* extract powders and dextrin) of 1-, 2-, and 4-L scale were 100 g, 500 g, and 1 kg, respectively.

After *S. miltiorrhiza* extract powders were mixed with dextrin by the three-dimensional motion mixer (ZNW-10, Beijing Kingslh Technology Development Co. Ltd., Beijing, China), the powders were dry-mixed at an impeller speed of 500 rpm and chopper speed of 500 rpm. Subsequently, the binder solution was added into the working vessel using a peristaltic pump (BT00-100M, Baoding Longer Precision Pump Co., Ltd., Baoding, Hebei, China). The granulation process was immediately stopped after the ethanol was added. At the end of granulation, the mixture was discharged and transferred into an electric heat drum wind drying oven at 60 °C for over 3 h.

A vibration screen with nine standard sieves (ZNS-300, Beijing Kingslh Technology Development Co. Ltd., Beijing, China) was used to separate the raw granules into three parts, including lumps, granules, and fine powders. Particles that could not pass through the 10 mesh sieves were defined as lumps. Those that could pass through 10 to 100 mesh sieves were defined as granules. Particles that could pass through 100 mesh sieves were defined as fine powders.

#### 2.2.2. Granule Size Distribution Determination

After thorough vibration screening, the weight of particles intercepted by each standard sieve was recorded to calculate the particle size. Given the particle size distribution, it was possible to acquire the cumulative particle size, including *D*_10_, *D*_50_, and *D*_90_. *D*_10_, *D*_50_, and *D*_90_ represent particle sizes corresponding to 10%, 50%, and 90% of the cumulative undersized distribution, respectively.

## 3. Methods

### 3.1. Plackett–Burman Experimental Design

To screen the granulation factors, the Plackett–Burman experimental design was conducted [34]. Nine granulation factors, including three-dimensional mixing time, dry mixing time, ethanol concentration, *S. miltiorrhiza* extract powder ratio, binder solution amount, binder addition time, impeller speed, chopper speed, and drying time were selected as independent factors, which varied at low and high levels (see Table 1). *D*_50_ was used as a dependent response.

Next, Design-Expert 8.0 software (Stat-Ease, Inc., Minneapolis, MN, USA) was applied to generate and evaluate the statistical experimental design involving 12 combinations. The matrix of Plackett–Burman design includes granulation factors and responses (see Section 4.2). 

### 3.2. Optimization by Response Surface Methodology

For the optimization of granulation conditions, 17 combinations were employed. *S. miltiorrhiza* extract powder ratio, E-binder solution amount, and H-chopper speed were selected as independent factors, which were varied at three levels (low, medium, and high) according to the Box–Behnken design (BBD). *D*_50_ was used as a dependent response. The other six factors were kept constant, as follows: three-dimensional mixing for 2 min, dry mixing for 1 min, addition of 90% ethanol for 60 s, impeller speed kept at 500 rpm, and drying at 60 °C for 2 h. Design-Expert 8.0 software was used for generation and evaluation of the BBD. 

By applying multivariate regression analysis, a fitted quadratic model was obtained from *D*_50_, given by the following equation:

Y = A_0_ + A_1_X_1_ + A_2_X_2_ + A_3_X_3_ + A_11_X_1_^2^ + A_22_X_2_^2^ + A_33_X_3_^2^ + A_12_X_1_X_2_ + A_13_X_1_X_3_ + A_23_X_2_X_3_

where Y is the arithmetic mean response and A_1_, A_2_, and A_3_ are the regression coefficients of the factors X_1_, X_2_, and X_3_, respectively [35,36].

The relevant model was obtained and validated. Response surface was established to estimate the optimal conditions. Thus, the value of *D*_50_ within the prescribed range could be predicted and validated by actual trials. 

### 3.3. Dimensional Analysis-Based Scale-Up 

Scale-up of the HSWG process was conducted under the guidance of dimensional analysis theory. Based on the results of screening and optimization of experiments, a U_5_ (5^2^) uniform design (see Table 2) was used to arrange the experiments at each scale (the working vessel from 1 to 4 L); the dimensionless parameters needed to be constant across different scales to obtain similar granules. 

It has been reported that a dimensionless group called the dimensionless spray flux (*Ψ_a_*) was used to quantify the effects of the most important process variables in the nucleation zone, such as liquid flow rate, binder drop size, and powder flux through the spray zone [37]. Furthermore, the other critical dimensionless factor is the dimensionless drop penetration time (*τ_p_*), which is the ratio of the rate at which the binder drops penetrate into the powder bed to the rate at which these drops are re-exposed to the spray [33]. *Ψ_a_* and *τ_p_* are defined as Equations (1)–(3). Various binder addition times could be calculated using Equations (1)–(5) for different solid ratios [14,31].

(1) $$\Psi_{a}=\frac{3\times \dot{V}}{2\times \dot{A}\times d_{d}}$$

(2) $$\dot{A}=v\times w$$

(3) $$\tau_{p}=\frac{t_{p}}{t_{c}}$$

(4) $$\frac{\Psi_{a1}}{\Psi_{a2}}=\frac{{\dot{V}}_{1}}{v_{1}}\times \frac{v_{2}}{\dot{V_{2}}}=\frac{V_{1}}{V_{2}}\times \frac{t_{2}}{t_{1}}\times \frac{r_{2}}{r_{1}}=1$$

(5) $$t_{2}=t_{1}\times \frac{r_{1}}{r_{2}}\times \frac{V_{2}}{V_{1}}$$

where $\dot{V}$ is the spray volume flux; *Ȧ* is the powder area flux; *d_d_* is the spray drop diameter; *v* is the particle velocity; *w* is the spray width; *t_p_* is the time it takes for a drop to fully penetrate the powder bed; and *t_c_* is the time it takes the exposed surface of powder to circulate back to the spray zone. The subscripts 1 and 2 represent processing at a 1- and 2-L scale, respectively; *t* is the binder addition time; *V* is the total volume of binder; and *r* is the radius of the impeller.

To keep the dimensionless parameters *Ψ_a_* and *τ_p_* constant, the ethanol addition times of different scales could be calculated according to our previous paper [14]. To assess the similarity of granules at three different scales, the granule size distributions were calculated and compared using the cosine method [14]. Moreover, the granule surface morphology was analyzed by SEM using a Quanta 250 (FEI company, Brno, Czech), as previously described by Ondřej et al. [38]. Briefly, after being sprayed with gold, appropriate amounts of *S. miltiorrhiza* granules were placed on the electron microscope platform with conducting resin. The parameters of SEM were set as follows: 180° scan; accelerating voltage, 15.0 kilovolt; spot, 4.0; test pattern ETD and magnification, 50–10,000. The flowability of granules was determined by the angle of repose.

## 4. Results

### 4.1. Preliminary Investigations of Granulation Factors and Response Selection 

The HSWG process was successfully used to convert powder to granules [39]. In considering the process from mixing through to wet granulation, many granulation factors should be controlled to ensure the quality of the final product, such as three-dimensional mixing time, dry mixing time, and ethanol concentration. Preliminary experiments were conducted to optimize the granulation factors. Granular material selection was investigated using dextrin, starch, α-lactose, sugar, and microcrystalline cellulose. The results showed that dextrin is a good granular material for forming stable granules. Moreover, preliminary investigations for granulation factor selection revealed that the *S. miltiorrhiza* extract powder ratio was no more than 50%, showing that there were no hard “balls” in the working vessel. In addition, to prevent caking, the granulation time should not be more than 2 min. To ensure that binder addition was controlled, the binder was delivered into the granulation bowl by a peristaltic pump with a tube instead of pouring. The inner diameter of this tube was 0.24 cm.

In the granulation process, a slightly larger granule size and better flowability could be regarded as the ideal granule properties. Therefore, granule size (*D*_50_) and flowability (angle of repose (AoR)) were selected as the responses.

### 4.2. Plackett–Burman Experimental Design

Plackett–Burman experimental design is commonly used to screen multiple factors affecting a particular response [40]. In the present study, the Plackett–Burman experimental design was used to evaluate nine independent factors regarding their effects on granule size by using Design-Expert 8.0.

The arrangement and results of the Plackett–Burman design are shown in Table 3. The data analysis of Table 3 (see also Table 4) showed that the model of *D*_50_ was significant, with *p* < 0.0001. Three granulation factors (D—*S. miltiorrhiza* extract powder ratio; E—binder solution amount; and H—chopper speed) had significant impacts on the *D*_50_ model, indicating these three factors were significant model terms. Furthermore, values of R-Squared, Adj R-Squared, and Pred R-Squared were more than 0.9, suggesting that this model performed well. Therefore, based on the screening results, we chose *S. miltiorrhiza* extract powder ratio, binder solution amount, and chopper speed to determine the main interactions and effects on granule size.

In this study, the model of *D*_50_ was significant (see Table 4), but the model of AoR was not. Hence, the ANOVA results of AoR are not displayed. In the subsequent optimization and validation assays, AoR was not performed and *D*_50_ was the only response.

### 4.3. Box–Behnken Experimental Design

The design matrix and *D*_50_ results of the Box–Behnken design are shown in Table 5. Furthermore, ANOVA was used to analyze the response surface reduced quadratic model. Table 6 indicates that the *S. miltiorrhiza* extract powder ratio (X_1_) and binder solution amount (X_2_) significantly affected the *D*_50_ value (*p* < 0.05), while the effect of chopper speed on the *D*_50_ had no statistical significance (*p* > 0.05). Moreover, the results of ANOVA for *D*_50_ confirmed that the prediction of *D*_50_ was successful using this model, indicated by the value of Prob > F, which was less than 0.05. In addition, no statistical significance was observed in the lack of fit, with *p* = 0.663, showing that any variation in the model was due to pure error. There was no significant interaction between the X_1_ and X_2_ (*p* = 0.0673) with respect to the *D*_50_.

The final equation in terms of coded factors is as follows:

*D*_50_ = 191.62 + 95.11 × X_1_ + 49.97 × X_2_ + 0.48 × X_3_ + 18.87 × X_1_ × X_2_ + 42.68 × X_1_^2^ + 15.39 × X_3_^2^.

From this equation, it could be concluded that these three granulation factors have a positive correlation with *D*_50_, even though not all their effects were statistically significant (*p* = 0.0673 for X_1_ × X_2_, *p* = 0.0512 for X_3_^2^).

**Figure 1:**
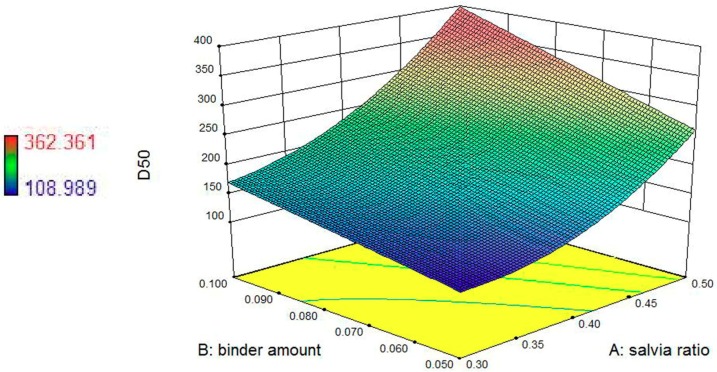
3D response surface showing the effects of the *S. miltiorrhiza* granule ratio and binder solution amount on *D*_50_.

**Figure 2:**
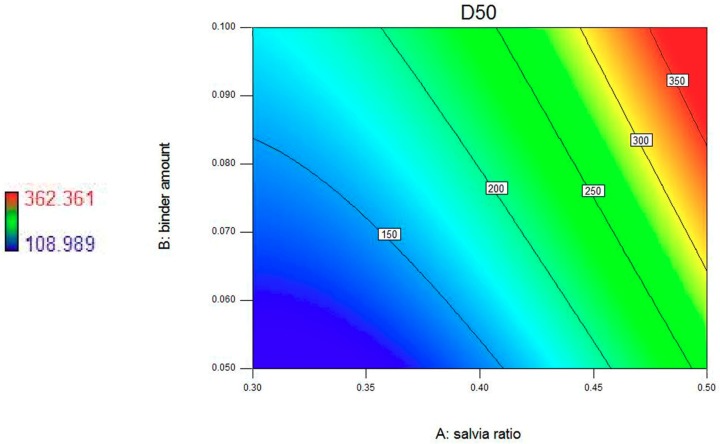
Contour plots showing effects of the *S. miltiorrhiza* extract powder ratio and binder solution amount on *D*_50_.

**Figure 3:**
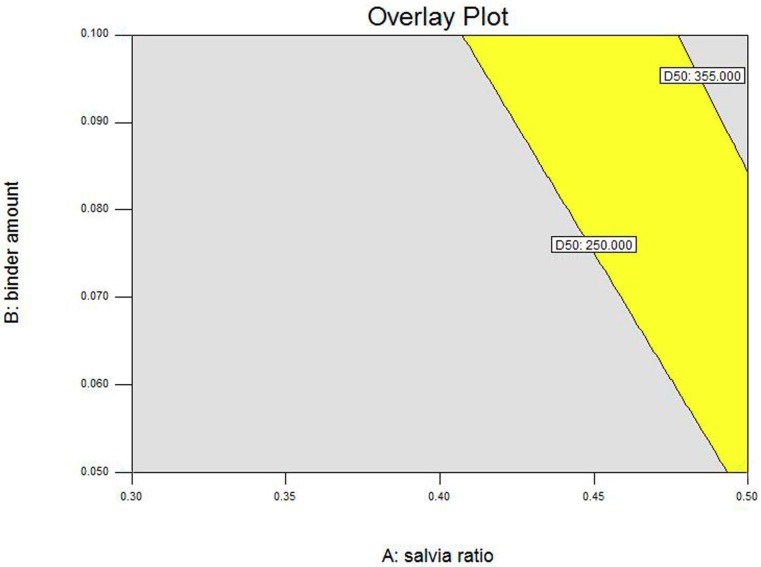
Design space of *D*_50_ for high shear wet granulation without confidence interval.

In our study, response surface and contour plots were also used to evaluate the effects of these granulation factors and their interactions on the response. Figure 1 shows the effects of the *S. miltiorrhiza* extract powder ratio and binder solution amount on *D*_50_, and contour plots reveal the curvature (see Figure 2). The results obtained, as seen in Figure 1 and Figure 2, indicate a positive correlation between *S. miltiorrhiza* extract powder ratio/binder solution amount and the *D*_50_ values. Therefore, the optimization goal of *D*_50_ was set between 250 and 355 μm. To visualize the appropriate operating range of the process variables, all possible combinations of these factors that met the optimization goal (250 μm < *D*_50_ < 355 μm) were extracted to build the design space of *D*_50_ (see Figure 3). 

**Figure 4:**
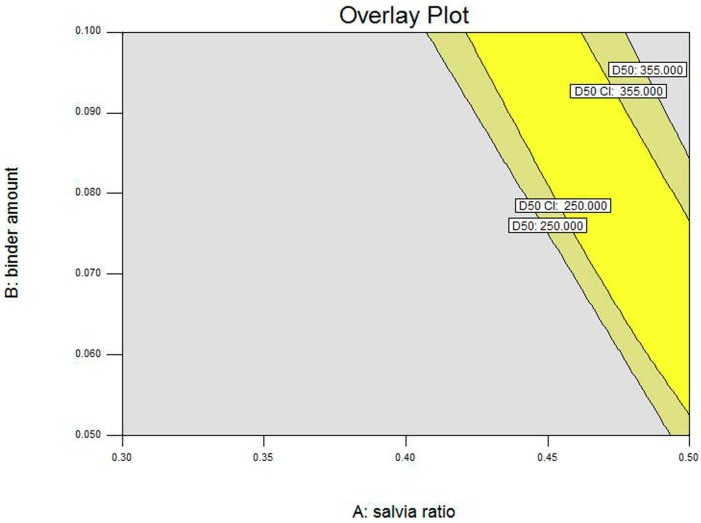
Design space of *D*_50_ for high shear wet granulation (HSWG) coupled with a 95% confidence interval.

Given that the boundary of the design space was uncertain, a 95% confidence interval (α = 0.05) was introduced to optimize the design space (see Figure 4). As shown in Figure 4, the bright yellow part was the optimized design space, and all points in this part achieved the optimization goal (250 μm < *D*_50_ < 355 μm). By contrast, the dark yellow part was the uncertain part of the design space, which revealed that there was a 5% chance that the prediction value of the points in this area might fail to meet the optimization goal (*D*_50_ < 250 μm or *D*_50_ > 355 μm).

Additionally, validation experiments were performed to evaluate the reliability and stability of the design space of *D*_50_. The results obtained from Table 7 indicate that under the process conditions of the point in the bright part of the design space, the optimization goal (*D*_50_ = 305.81, 292.26, 298.45, 300.45, and 303.20 μm) was achieved. However, the points in the dark part of the design space did not meet the optimization goal (*D*_50_ = 508.94, 468.47, and 420.56 μm). 

### 4.4. Similarity of Granule Size Distribution

**Figure 5:**
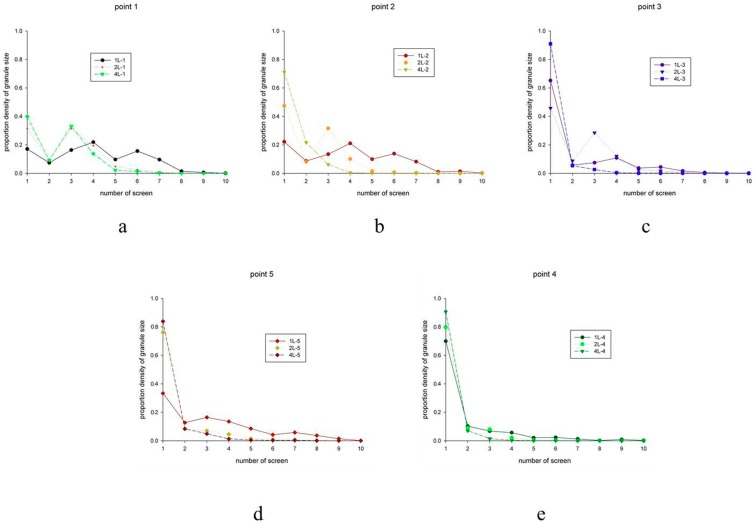
Probability density distributions of five points at three different scales; (**a**–**e**) experiment points 1–5 of the uniform design U_5_ (5^2^). For each figure, different symbols represent 1, 2, and 4 L, respectively.

The granule sizes of five points at three different scales are shown in Figure 5. Similar size distribution patterns were observed in the probability density distributions of granule size at 1-, 2-, and 4-L scales. Among the particle samples, lumps (>850 μm) and fine powder (<180 μm) comprised a small proportion, and the size of most of the granules fell between 180 and 850 μm. The cosine values of granule size distributions at 2- and 4-L scales were calculated for comparison with the granule size distribution of the 1-L granulation scale (see Table 8). The results indicate that all cosine values of 2- and 4-L scales were greater than 0.8, suggesting that the probability granule size distribution curves of both 2- and 4-L scales matched the distribution curves of the 1-L scale well.

### 4.5. Granule Morphology

**Figure 6:**
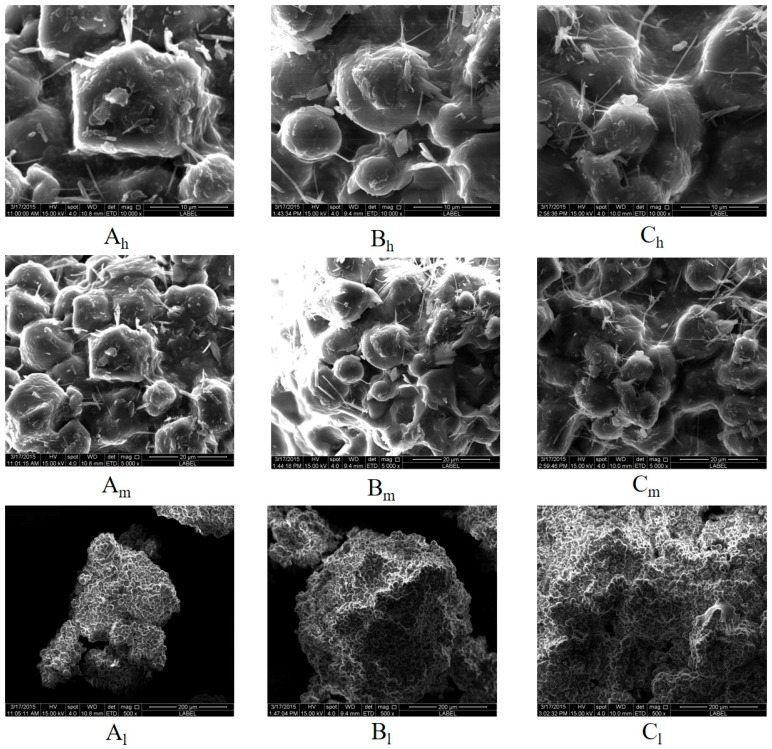
Scanning electron microscopy images of *S. miltiorrhiza* granules obtained at the same processing point but at different scales of HSWG. Critical process parameters (CPPs) of point 1 were as follows: three-dimensional mixing for 2 min, dry mixing for 1 min, addition of 90% ethanol for 60 s, impeller speed kept at 500 rpm, chopper speed kept at 1000 rpm, and drying at 60 °C for 2 h. The proportion of *S. miltiorrhiza* extract was 40%. The proportion of 90% ethanol was 10%. The addition times for 1-, 2-, and 4-L scales were 60, 88, and 140 s, respectively. Labels A, B, and C represent 1-, 2-, and 4-L scales, respectively. “h”, “m”, and “l” in the figure represent high (10,000×), medium (5000×), and low (200×) magnifications, respectively.

Figure 6 shows the SEM images of *S. miltiorrhiza* granules produced at the same processing point but from different scales of HSWG, indicating *S. miltiorrhiza* granules produced from 1-, 2-, and 4-L scales of HSWG had similar morphologies and surface structures. Moreover, the high- and medium-magnification images (Figure 6: A_h_, B_h_, C_h_, A_m_, B_m_, C_m_) show that crystal structures were observed on the rough surface of *S. miltiorrhiza* granules produced at different scales. However, the low-magnification images (Figure 6: A_l_, B_l_, and C_l_) reveal the irregular edges and bumpy surfaces of *S. miltiorrhiza* granules at 1-, 2-, and 4-L scales.

### 4.6. Granule Flowability

The results for the angle of repose of *S. miltiorrhiza* were obtained. Dextrin granules are shown in Table 9; according to the European Pharmacopoeia 9.8 [41], particles could be divided into seven groups in terms of flowability and value of the angle of repose (see Table 10). For each experiment point, all values of the angle of repose were between 30° and 32°, indicating that the flowability of *S. miltiorrhiza* granules produced at 1-, 2-, and 4-L scales were good. Based on the classification standard of flowability, these granules should be classified into the same grade. SPSS software (IBM SPSS software, Armonk, NY, USA) was utilized to test the significance of differences in flowability of *S. miltiorrhiza* granules at different scales. Paired sample *t*-tests were performed between the values of the angle of repose from 2- and 4-L scales and those of the 1-L scale. All *p*-values were greater than 0.4 with respect to the 1-L data, suggesting there was no significant difference in flowability among granules at the three scales. 

## 5. Discussion

QbD is a comprehensive approach targeting all phases of drug discovery, manufacturing, and delivery [42,43,44]. QbD consists of many elements, such as quality target product profile (QTPP), critical quality attributes (CQAs), critical process parameters (CPPs), risk assessment, design space, control strategy, product lifecycle management, and continual improvement. CPPs are process parameters whose variability has an impact on critical quality attributes and should therefore be monitored or controlled to ensure that the process achieves the desired quality. Moreover, CQAs are physical, chemical, biological, or microbiological properties or characteristics that should be within an appropriate limit, range, or distribution to ensure the desired quality of a product [7]. Therefore, screening designs should firstly be applied to identify the CPPs from the quantitative impact on the CQA [45]. Next, optimization designs should be used to allow optimal set points for the design or control space to be identified in order to target desired CQA values [46]. Finally, validation experiments should be conducted to confirm whether the design space meets the optimization goal. As a result, process robustness and operation flexibility could be improved using the QbD approach [47].

In the HSWG process, the quality attributes of the manufactured granules are determined by the performances of the raw powder for feed and binder liquid, as well as by the equipment configuration and process operating conditions. In the manufacturing process for chemical drug preparation, and especially for granules, tablets, and other solid dosage forms, a water-based polymer solution is often used as the binder solution. For example, hydroxy propyl methyl cellulose (HPMC) and polyvinyl pyrrolidone (PVP) are commonly used in the chemical drug granulation process [48,49]. However, most APIs of chemical drugs in solid dosage forms are obtained through chemical synthesis or biosynthesis [50]. The viscosities of these APIs are very poor. Thus, a sticky binder solution is added to the granulation process to provide the extra viscosity that is needed. One of the most common problems in the granulation process is how to select a proper binder solution that provides sufficient viscosity to bond the powder together. By contrast, APIs of traditional Chinese medicines (TCMs) in solid dosage forms are often from plant extracts, such as in the case of *S. miltiorrhiza* extract. These plant extracts are very sticky and actually hinder the granulation process. Hence, to improve this unsatisfactory condition, a binder solution such as ethanol is needed to mitigate the extra viscosity [51]. Thus, in this study, ethanol of different concentrations was selected as the binder solution.

Granule size, as one of the characteristics of granules, is influenced by numerous granulation factors. Notably, many studies have shown that impeller speed could significantly affect granule size in most cases [34]. In a system of calcium carbonate (feed powder)–polyethylene glycol (binder solution), however, Rahmanian reported [52] that granule size distribution did not seem to be markedly affected by impeller speed. Moreover, Wang et al. [53] indicated that the effect of impeller speed on granule properties was dependent on the starting material system, and that chopper speed from 1200 to 3600 rpm had a consistent influence on all formulations. Our previous study also reported that [54] the impeller speed did not have a significant impact on granule size in the system of microcrystalline cellulose –water. In line with these reports, our results did not demonstrate any obvious changes in granule size when the impeller speed was increased. This may be attributed to the features of our starting material system (*S. miltiorrhiza* extract).

Additionally, it has been reported that wet massing time also has a statistically significant impact on granule size [34]. However, in our preliminary experiment, no granules were formed when wet massing time was set from 1 to 3 min and, instead, many hard balls formed that were outside the range of the desired granule size. Considering the API we used, the *S. miltiorrhiza* extract was very sticky. Although ethanol was selected as the binder solution to induce and reduce the viscosity of the system, it was not enough to solve this problem. As such, wet massing time was not incorporated in the experimental design. In the MCC–water system of our previous work [14,54], binder amount and wet massing time were both significant factors affecting granule quality. Thus, in follow-up experiments, we will attempt to match the sticky API by using other excipients and binders.

A variety of QbD approaches have been applied by the researchers, including the use of design of experiments to screen and optimize process conditions with high efficacy, speed, and accuracy [55,56,57]. Plackett–Burman experimental design is commonly used to screen the most important factors early in the pharmaceutical experimentation phase to generate reliable and more manageable sets of combinations, as well as to indicate how each factor affects the response [58]. Box–Behnken design, one of the designs of the response surface methodology, has been applied in the optimization of pharmaceutical processes because of its reasonable design and excellent outcomes. In our present study, Plackett–Burman experimental design was employed to screen the granulation factors of *S. miltiorrhiza* granules in the HSWG process. The results showed that three granulation factors (salvia ratio, binder amount, and chopper speed) were identified from nine granulation parameters and remarkably affected the value of *D*_50_. This suggests that design is a very useful tool to screen the main effects when a large number of factors are to be evaluated in the HSWG process. Moreover, a three-level Box–Behnken design was used in the present study, and the optimal conditions of salvia ratio and binder amount were determined. Notably, a strong agreement was observed among the *D*_50_ predicted by the quadratic polynomial model and the validation experimental results, ANOVA of quadratic polynomial model, and value of the lack of fit, suggesting that the accuracy and general ability of this model were very good. Hence, the results obtained from the Plackett–Burman design and Box–Behnken design indicate that these two methods could be effective screening and optimization strategies in the HSWG process.

The significant rate processes for HSWG consist of wetting and nucleation, coalescence and growth, and breakage [31]. The nucleation regime map approach represents an intermediate level modeling approach for the application of QbD to the formulation design and scale-up of geometrically similar granulators, and was proposed and investigated for wetting and nucleation processes in the present study [33]. For scale-up of the HSWG process, dimensionless parameters such as *Ψ_a_* and *τ_p_* should be kept constant across different scales so that the microenvironment of granule nucleation and growth are controlled and similar granule properties can be obtained [59]. In the present study, *Ψ_a_* and *τ_p_* remained the same for each point of 1-, 2-, and 4-L scales and produced similar *S. miltiorrhiza* granules, as indicated by their nearly identical properties. 

In this study, the similarity of granules produced by three different scales were evaluated from three aspects, including granule size distribution, granule surface morphology, and flowability. It has been reported that sieve analysis has been widely used to form granulation distribution curves according to the weight of granules trapped by sieves with different mesh sizes [60]. Moreover, granule size was detected by the distribution curves. The results show that a good fit was observed between two different granule size distribution curves, revealing that the granules produced by these two scales had similar granule size distributions. Moreover, the appearance of granules gives an indication of granule surface morphology, with similar surface morphologies providing a similar look and feel [14]. In addition, flowability is a comprehensive property of powder and granules. It can be affected by many factors, such as particle size, uniformity, density, porosity, surface area, stickiness, and so on [61,62]. Granule flowability has an impact on subsequent operations in solid preparation production, especially tableting, and poor granule flowability may cause variability in tablet quality [63,64]. Parallel flowabilities of granules mean that granules produced at various scales have almost the same flow properties. Our results show that granules produced by three different scales had similar granule size distributions and morphologies as well as parallel flowability, suggesting that the regime map approach could be successfully employed to scale-up the HSWG process.

## 6. Conclusions

Collectively, our study used QbD approaches to comprehensively evaluate the HSWG process using *S. miltiorrhiza* granules. It was found that the *D*_50_ of *S. miltiorrhiza* granules was obviously influenced by the salvia ratio, binder amount, and chopper speed. The scale-up of the HSWG process could be performed rationally using a nucleation regime map approach. This study provides a better understanding of the HSWG process through QbD approaches using *S. miltiorrhiza* granules. 

## Figures and Tables

**Table 1:** Granulation factors and response variables for the Plackett–Burman experiment design.

Factor Abbreviation	Granulation Factors	Level Used	
		Low	High
A	Three-dimensional mixing time	2 min	20 min
B	Dry mixing time	1 min	6 min
C	Ethanol concentration	60%	90%
D	*Salvia miltiorrhiza* extract powder ratio	20%	50%
E	Binder solution amount	0.05	0.10
F	Binder addition time	30 s	90 s
G	Impeller speed	300 rpm	1000 rpm
H	Chopper speed	450 rpm	2900 rpm
I	Drying time	1 h	24 h

**Table 2:** Experimental schedule of the uniform design U_5_ (5^2^).

Run	Ratio of *S. miltiorrhiza* Extract Powder (%)	Ethanol Amount (%)
1	40.0	10.0
2	42.5	8.75
3	45.0	7.50
4	47.5	6.25
5	50.0	5.00

**Table 3:** Plackett–Burman design matrix and results for screening of various granulation factors. AoR was short for angle of repose, which was an index of granule flowability.

Run	A	B	C	D	E	F	G	H	I	*D*_50_ (μm)	AoR (°)
1	20	6	90	20	0.05	30	1000	450	24	103.4	31.95
2	20	1	60	20	0.1	30	1000	1500	1	142.7	34.99
3	20	6	60	20	0.05	90	300	1500	24	54.00	25.07
4	2	6	60	50	0.1	30	1000	1500	24	324.1	28.28
5	2	1	60	50	0.05	90	1000	450	24	323.0	34.08
6	20	1	90	50	0.1	30	300	450	24	443.7	33.33
7	2	1	60	20	0.05	30	300	450	1	84.90	24.16
8	20	6	60	50	0.1	90	300	450	1	433.2	34.31
9	2	6	90	50	0.05	30	300	1500	1	321.8	32.92
10	2	6	90	20	0.1	90	1000	450	1	231.4	33.14
11	2	1	90	20	0.1	90	300	1500	24	119.5	32.78
12	20	1	90	50	0.05	90	1000	1500	1	229.7	34.74

**Table 4:** ANOVA results for *D*_50_ of Plackett–Burman design.

Source	Sum of Squares	df	Mean Square	*F*-Value	*p*-Value Pro > F
Model	1.96 × 10^5^	3	64,202.41	64.72	<0.0001
D	1.496 × 10^5^	1	1.496 × 10^5^	150.76	<0.0001
E	27,813.19	1	27,813.19	28.04	0.0007
H	15,238.69	1	15,238.69	15.36	0.0044
Residual	7935.97	8	992.00		
Cor Total	2.005	11			
SD		31.50	R-Squared		0.9604
Mean		234.29	Adj R-Squared		0.9456
Coefficient of variation (CV)%		13.44	Pred R-Squared		0.9110
PRESS		17,855.94	Adeq Precision		21.493

**Table 5:** Box–Behnken experimental design matrix for optimizing granulation factors.

Run	X_1_: *S. miltiorrhiza* Extract Powder Ratio	X_2_: Binder Solution Amount	X_3_: Chopper Speed	*D*_50_ (μm)
1	40%	0.050	500	153.60
2	50%	0.100	1000	362.36
3	40%	0.100	1500	243.05
4	40%	0.075	1000	191.82
5	50%	0.075	500	859.23
6	40%	0.100	500	254.16
7	30%	0.075	500	159.40
8	50%	0.050	1000	260.27
9	40%	0.075	1000	191.37
10	40%	0.050	1500	152.04
11	50%	0.075	1500	359.07
12	40%	0.075	1000	168.45
13	30%	0.075	1500	161.14
14	40%	0.075	1000	208.31
15	30%	0.100	1000	182.30
16	30%	0.050	1000	108.99
17	40%	0.075	1000	188.61

**Table 6:** ANOVA results for response surface reduced quadratic model.

Source	Sum of Squares	df	Mean Square	F Value	*p*-Value Prob > F
Model	74,828.71	6	12,471.45	75.49933	<0.0001 *
X_1_	34,585.91	1	34,585.91	209.3753	<0.0001 *
X_2_	13,813.87	1	13,813.87	83.62602	<0.0001 *
X_3_	1.472887	1	1.472887	0.008917	0.9268
X_1_ × X_2_	714.2027	1	714.2027	4.323621	0.0673
X_1_^2^	20,989.53	1	20,989.53	127.0659	<0.0001 *
X_3_^2^	834.609	1	834.609	5.052533	0.0512
Residual	1486.676	9	165.1862		
Lack of fit	680.3145	5	136.0629	0.67	0.663
Pure error	806.3618	4	201.5904		
Cor total	76,315.38	15			
SD		12.85248	R-Squared		0.980519
Mean		209.0576	Adj R-Squared		0.967532
CV%		6.147817	Pred R-Squared		0.712246
PRESS		21,960.06	Adeq Precision		30.27463

Asterisks denote most significant factors and interaction effects (*p*-value < 0.05).

**Table 7:** Validation experiment results.

Run	X_1_: *S. miltiorrhiza* Extract Powder Ratio	X_2_: Binder Solution Amount	X_3_: Chopper Speed (rpm)	*D*_50_ (μm)
1	46%	0.086	933	305.81
2	44%	0.100	719	508.94
3	47%	0.068	1367	468.67
4	43%	0.100	1300	292.26
5	44%	0.095	1200	298.45
6	45%	0.090	1100	300.45
7	46%	0.085	1000	303.20
8	47%	0.080	1300	420.56

**Table 8:** Cosine values of probability density distribution of granule size at 2- and 4-L process scales compared with that at a 1-L scale.

Scale (L)	1	2	3	4	5
2	0.8438	0.8753	0.9042	0.9971	0.8546
4	0.8690	0.8336	0.9799	0.9905	0.8253

**Table 9:** Angle of repose of *S. miltiorrhiza* granules at different scales.

No.	Average Value (°)	SD (°)	RSD (%)
1 L-1	30.48	0.27	0.90
2 L-1	30.58	0.58	1.90
4 L-1	30.39	0.28	0.93
1 L-2	30.69	0.20	0.64
2 L-2	30.72	0.27	0.89
4 L-2	30.71	0.22	0.71
1 L-3	30.45	0.26	0.85
2 L-3	30.65	0.26	0.85
4 L-3	30.37	0.18	0.58
1 L-4	31.14	0.57	1.85
2 L-4	31.47	0.37	1.17
4 L-4	31.17	0.72	2.32
1 L-5	32.50	0.26	0.79
2 L-5	32.38	0.72	2.23
4 L-5	32.73	0.60	1.82

**Table 10:** Flow properties and corresponding values of angle of repose.

Flow Property	Value of Angle of Repose (°)
Excellent	25–30
Good	31–35
Fair (aid not needed)	36–40
Passable (may hang up)	41–45
Poor	46–55
Very poor	56–65
Very, very poor	>66
